# Altered cross-bridge properties in skeletal muscle dystrophies

**DOI:** 10.3389/fphys.2014.00393

**Published:** 2014-10-14

**Authors:** Aziz Guellich, Elisa Negroni, Valérie Decostre, Alexandre Demoule, Catherine Coirault

**Affiliations:** ^1^Service de Cardiologie, Hôpital Henri Mondor, University Paris-Est CréteilCréteil, France; ^2^Equipe 8, Institut National de la Santé et de la Recherche MédicaleCréteil, France; ^3^UMRS 974, Institut National de la Santé et de la Recherche MédicaleParis, France; ^4^UM 76, Université Pierre et Marie Curie, Sorbonne UniversitésParis, France; ^5^UMR 7215, Centre National de la Recherche ScientifiqueParis, France; ^6^Institut de MyologieParis, France; ^7^Assistance Publique-Hopitaux de Paris, Service de Pneumologie et Reanimation MedicaleParis, France

**Keywords:** myosin, cross-bridge kinetics, skeletal muscle, muscle dystrophy, myopathies

## Abstract

Force and motion generated by skeletal muscle ultimately depends on the cyclical interaction of actin with myosin. This mechanical process is regulated by intracellular Ca^2+^ through the thin filament-associated regulatory proteins i.e.; troponins and tropomyosin. Muscular dystrophies are a group of heterogeneous genetic affections characterized by progressive degeneration and weakness of the skeletal muscle as a consequence of loss of muscle tissue which directly reduces the number of potential myosin cross-bridges involved in force production. Mutations in genes responsible for skeletal muscle dystrophies (MDs) have been shown to modify the function of contractile proteins and cross-bridge interactions. Altered gene expression or RNA splicing or post-translational modifications of contractile proteins such as those related to oxidative stress, may affect cross-bridge function by modifying key proteins of the excitation-contraction coupling. Micro-architectural change in myofilament is another mechanism of altered cross-bridge performance. In this review, we provide an overview about changes in cross-bridge performance in skeletal MDs and discuss their ultimate impacts on striated muscle function.

## Introduction

**Figure 1:**
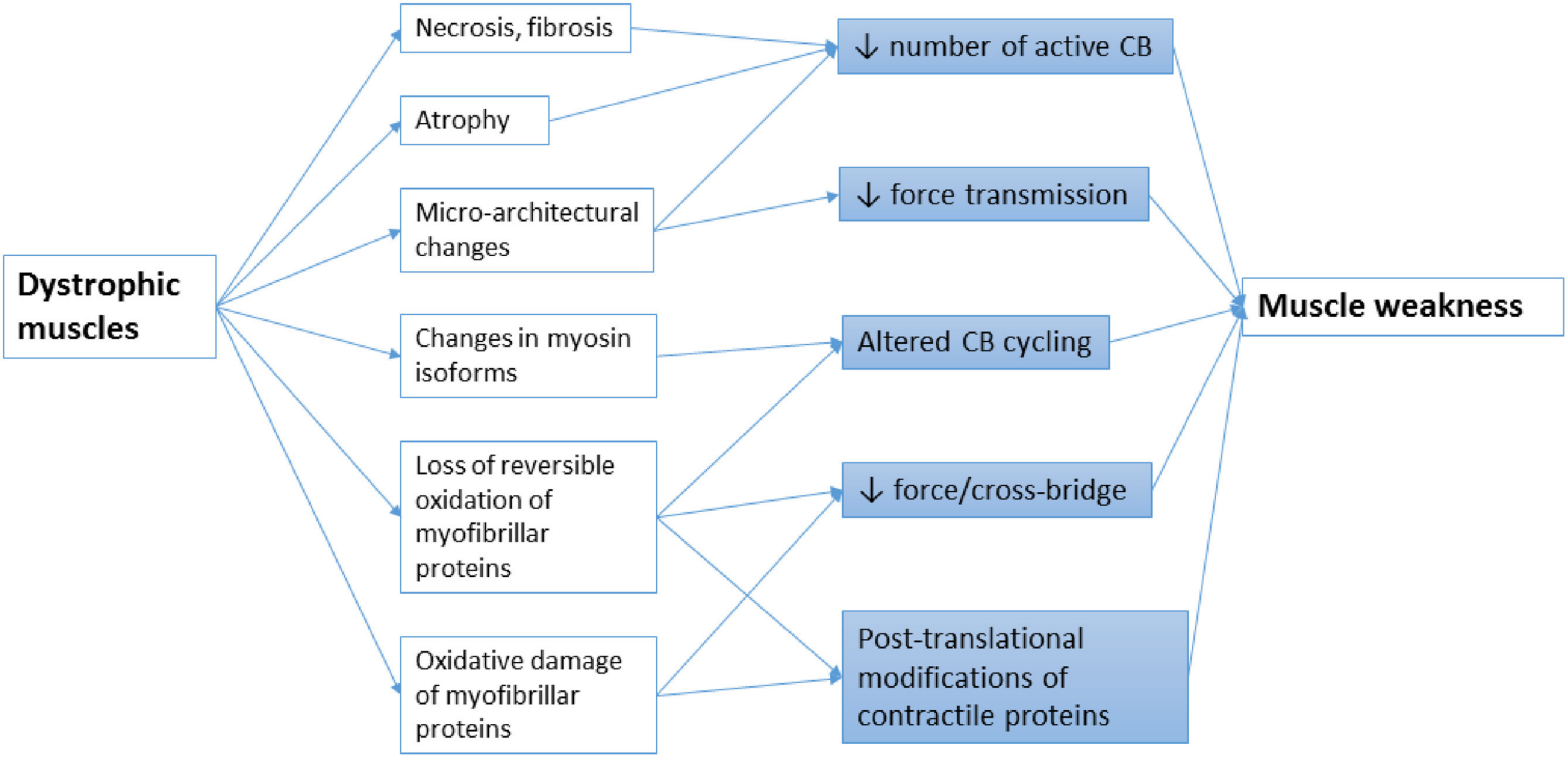
**Schematic representation of the main mechanisms leading to muscle weakness in MDs**. Altered CB cycle in muscular dystrophy can arise from multiples causes including progressive loss of muscle tissue due to necrosis, fibrosis, atrophy, or oxidative damage, micro-architectural alterations in myofilaments, altered redox regulation or pathological changes in contractile protein isoforms. These abnormalities in turn directly reduce the number, the force, the kinetics of active CBs or the transmission of force produced by the CBs.

Muscular dystrophies (MDs) are a group of more than 30 clinical and molecular heterogeneous genetic disorders that cause progressive degeneration of the skeletal muscle fibers. They are characterized by severe muscle weakness that generally affects limb, axial, and/or facial muscles to a variable extent. The age of onset, severity and rate of progression greatly vary in the different forms of MD. The primary cause of various forms of MDs is an individual mutation in genes encoding a wide variety of proteins, including extracellular matrix (ECM) proteins, transmembrane, and membrane-associated proteins, cytoplasmic proteases and nuclear proteins. Detailed classification and list of causative genes in MD have been recently reviewed (Cohn and Campbell, 2000; Flanigan, 2012; Kaplan and Hamroun, 2012; Mercuri and Muntoni, 2013). Although still incompletely understood, considerable progress has been now made to reveal the pathophysiological mechanisms in MDs. It has been shown that most MDs share common pathologic features, such as altered Ca^2+^ homeostasis, infiltration of muscle tissue by inflammatory immune cells, accretion of proinflammatory and profibrotic cytokines, activation of proteolytic enzymes, metabolic/mitochondrial alterations, intracellular accumulation of reactive oxygen species (ROS) production, and/or defective autophagy, which can contribute to muscle wasting. At the earliest stages of the disease, reduced myofibrillar protein content can occur in apparent uninjured fibers, secondary to an imbalance between protein synthesis and proteolysis (McKeran et al., 1977; Warnes et al., 1981). Then, successive rounds of degeneration and regeneration lead to fibrosis and fatty replacement of muscle tissue and in turn reduce the number of potential cross-bridges (CBs) generating force (Figure 1). In addition, functional changes in the CB properties may contribute to muscle weakness in distinct types of muscle disease. It is well-established that shift in the relative myosin isoform expression is associated with modifications of the kinetics of actomyosin interactions (Bar and Pette, 1988; Schiaffino et al., 1989; Schiaffino and Reggiani, 1996). However, there are also increasing evidences that CB properties can change with no change in myosin isoform content (Coirault et al., 2002; Canepari et al., 2009), suggesting that post-translational modifications of contractile proteins have significant role in muscle weakness (Figure 1). The present review focuses on the changes in the function of contractile proteins and in CB performance that may occur in the context of skeletal muscle dystrophies (MDs) and their contribution to the pathophysiological mechanisms of these diseases.

## Cyclical interactions of actin with myosin

Contractile force of striated muscle is produced within the half-sarcomere—the functional contractile unit—by the cyclical interactions of actin filament with myosin molecule, the muscle molecular motor. These cyclical interactions are controlled by membrane-located mechanisms that trigger intracellular raise of Ca^2+^and mechanisms inside the sarcomere itself. In skeletal muscle, sarcomeric mechanisms mainly involve the Ca^2+^-controlled conformational change of the regulatory proteins troponins and tropomyosin (Tm), the functional properties of myosin, and strong cooperative interaction between neighboring troponin-Tm unit along the actin filament (Gordon et al., 2000).

In resting muscle, intracellular Ca^2+^ concentration ([Ca^2+^]_i_) is low and the C-terminal domain of troponin I immobilizes the Tm in a position that prevents the binding of myosin to actin (Galinska-Rakoczy et al., 2008). Upon membrane depolarization, Ca^2+^ is released from the sarcoplasmic reticulum and binds to troponin C, initiating azimuthal movement of Tm around the actin-containing thin filament. This in turn exposes the sites on actin on which the myosin heads can attach (Kress et al., 1986; Geeves et al., 2005). In the conventional view, myosin binding is solely determined by the Ca^2+^ transient causing structural changes in the thin filament. However, numerous studies have reported Ca^2+^-induced structural change in the myosin-containing thick filaments before myosin binding to the thin filament (Huxley et al., 1982; Lowy and Poulsen, 1990; Yagi, 2003; Reconditi et al., 2011). This has led to the proposal of a modified model of muscle activation in which fast coordinated changes in the structures of both thick and thin filaments follow concomitantly upon the rise in intracellular free Ca^2+^ concentration (Reconditi et al., 2014).

**Figure 2:**
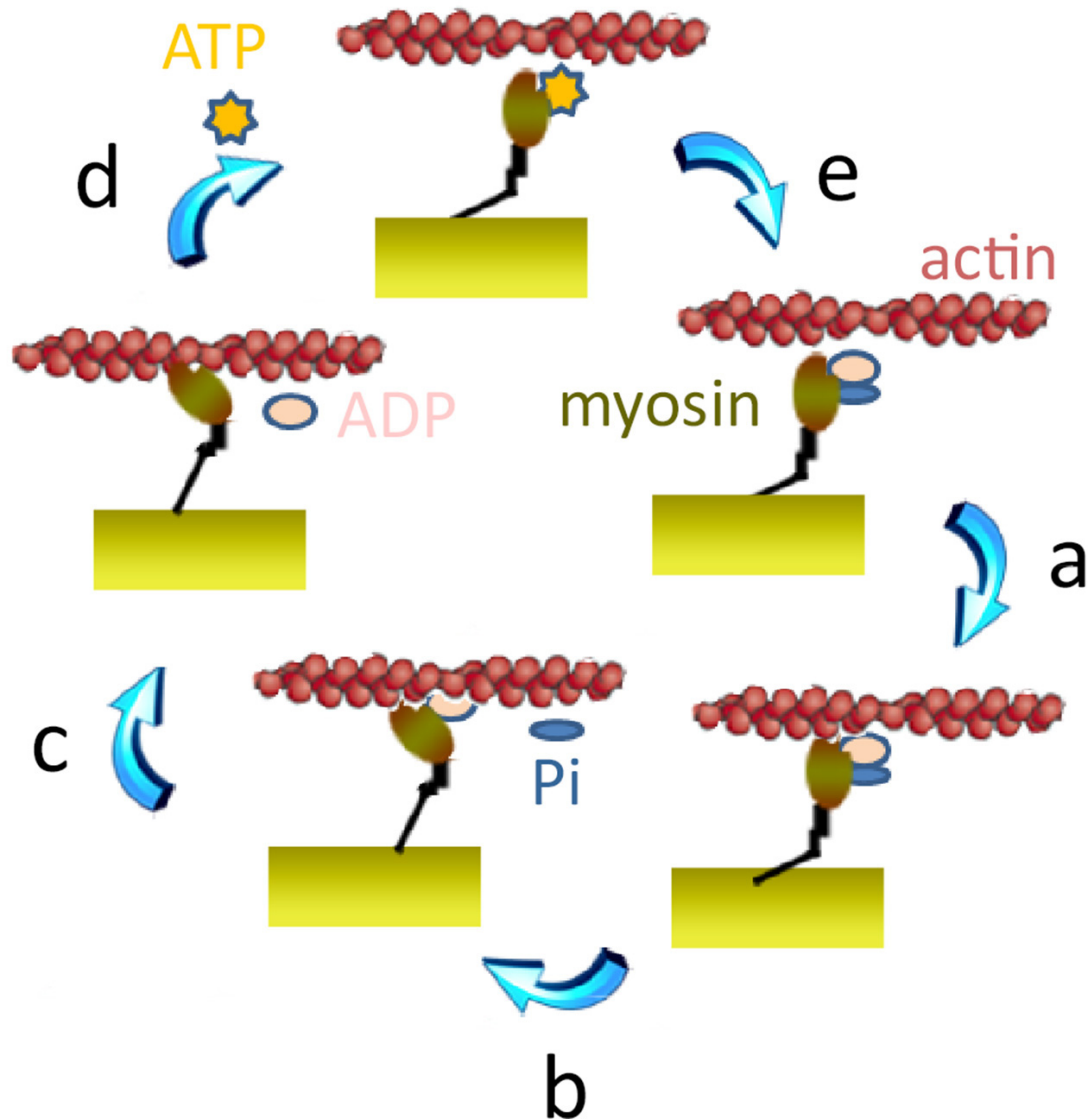
**The CB cycle**. Step a: attachment of myosin to actin. This step begins with the “weak binding” of actin (in magenta) to the myosin head (in brown). A stereospecific interaction between actin and myosin leads to strong binding of actin to myosin. Step b: power stroke. The loss of inorganic phosphate (Pi) is assumed to trigger the onset of the power stroke and a reversal of the previous conformational bent. Step c: ADP release step. The power stroke induces reopening of the nucleotide binding pocket and ADP release. Step d: ATP binding. ATP can then rapidly bind to myosin which dissociates from actin. Step e: ATP hydrolysis. ATP is then rapidly hydrolyzed to ADP and Pi.

Myosin head binding to actin occurs first in a low binding, pre-force-generating state (Figure 2, step a). At this state, the CB has already hydrolyzed the adenosine 5′-triphosphate (ATP), but the products adenosine 5′-diphosphate (ADP) and inorganic phosphate (Pi) are still bound to the myosin head (Lymn and Taylor, 1971; Pate and Cooke, 1989). Then, the CB goes through the power stroke (Figure 2, step b), during which the myosin head can generate a force of several piconewtons or an axial displacement of the actin filament toward the center of the sarcomere of 5–10 nm *in vitro* (Molloy et al., 1995; Veigel et al., 1998; Mehta et al., 1999; Reconditi et al., 2011) or 8–13 nm *in situ* (Reconditi et al., 2004). There are evidences that force generation precedes the Pi release (Dantzig et al., 1992; Caremani et al., 2008). Subsequent steps involve the release of ADP (Figure 2, step c), and the binding of ATP that rapidly dissociates the actomyosin complex (Figure 2, step d). When unbound from actin, myosin hydrolyzes ATP and reverses the power stroke, thus returning to its original position and allowing a new cycle to start (Eisenberg and Greene, 1980; Steffen and Sleep, 2004) (Figure 2, step e). Thus, at the molecular level, skeletal myosin is a molecular motor that transduces chemical energy produced by the hydrolysis of one ATP molecule into mechanical work (Huxley, 1957; Huxley and Simmons, 1971; Lymn and Taylor, 1971; Eisenberg et al., 1980; Pate and Cooke, 1989; Gordon et al., 2000). Skeletal MDs may reduce the number of active CBs producing force but may also potentially affect various steps of the cyclical interactions of actin with myosin, thus reducing the unitary force produced by each CB and/or modifying the CB kinetics.

## Changes in CB kinetics related to MD-induced shift in myosin isoform

The events in the CB cycle are essentially the same for all muscle myosins, but the kinetics of acto-myosin interaction varies widely among myosin heavy chain (MyHC) isoforms. Although the physiological differences between fast and slow skeletal muscle fibers depend on more than just differences in MyHC isoforms, it is well established that MyHC isoforms are a major determinant of the large variability in contractile and energetic properties of muscle fibers (Pette and Staron, 1990; Bottinelli et al., 1994a,b). The existence of several MyHC isoforms differentially distributed in various fibers makes MyHCs useful to study fiber heterogeneity and plasticity in mammalian muscles. In the adult skeletal muscle, four MyHC isoforms can be expressed, namely the slow MyHC -1 and the fast MyHC -2A, MyHC-2X, and MyHC-2B, coded respectively by the *Myh7, Myh2, Myh1*, and *Myh4* genes (Bar and Pette, 1988; Schiaffino et al., 1989; Schiaffino and Reggiani, 1996).

Changes in the proportion of fast relative to slow fiber types are frequently observed in muscular dystrophy, and may result either from a reduced muscular activity in affected patients or from a physiopathological consequence of the gene mutation. The pattern of wasted muscle and affected fibers is highly variable among the types of MDs. Preferential involvement in fast type 2 muscle fibers is a common feature in Duchenne Muscular Dystrophy (DMD) (Webster et al., 1988), with early disappearance of muscle fibers expressing MyHC-2X transcripts (Pedemonte et al., 1999). Likewise, slow type 1 muscle fiber predominance has been reported in patients with facioscapulohumeral muscular dystrophy (FSHD) (Celegato et al., 2006), dysferlinopathy (Fanin and Angelini, 2002) and congenital muscular dystrophy type Ullrich (Schessl et al., 2008), suggesting a selective loss of fast-twitch/type 2 muscle fibers (D'Antona et al., 2007) or an active fiber typing conversion process (Schessl et al., 2008; De La Torre et al., 2009). This preferential involvement of the fastest fiber type leads to a fast to slow shift among fast MyHC isoforms in the dystrophic muscles (Stedman et al., 1991; Petrof et al., 1993; Coirault et al., 1999), which may represent an adaptive response that would tend to preserve the economy of force contraction. However, fiber-type composition has been shown to be differently affected in other MDs. Hypertrophy of type 2 fibers without type 1 fiber atrophy have been found in biopsies of patients with laminopathies (Kajino et al., 2014), whereas MyHC-2a fibers are markedly atrophied in affected cricopharyngeal muscle of oculopharyngeal muscular dystrophy patients (Gidaro et al., 2013). How mutations responsible for MDs differently affect myofiber-type specification and/or MyHC deserve further studies. In addition to adult myosin isoform remodeling, dystrophic fiber can express developmental embryonic and neonatal MyHCs, coded by *Myh3* and *Myh8*, respectively (Wieczorek et al., 1985; Sartore et al., 1987). Such developmental or neonatal MyHC-expressing fibers can either be regenerating fibers or fibers that expressed inappropriate and immature MyHC, as has been observed in some congenital myopathies (Sewry, 1998).

Importantly, the different MyHC isoforms display large functional differences regarding the actin-activated ATPase activity of myosin, the rate of ADP release from acto-myosin during the time of attachment, and the velocity with which they can move actin. This results in large variability in contractile, thermodynamic, and kinetic coupling according to the fiber type (Barany, 1967). For instance, fibers containing MyHC-1 have nearly threefold slower ATPase and lower tension cost than fibers with MyHC-2X, while fibers with MyHC-2A are intermediate (Stienen et al., 1996). Therefore, an increased proportion of the slow MyHC isoforms would tend to improve muscle efficiency and may represent an adaptive response at least in some MDs. An opposite response, namely a slow-to-fast shift with preferential atrophy of slow fiber, accelerates the time cycle, reduces muscle efficiency (Coirault et al., 1999) and thus may contribute to the exercise intolerance in DMD patients. MyHC shift may also impact on CB recruitment, given that attachment of fast myosin involves a CB-mediated facilitation mechanism absent in slow MyHC isoforms (Galler et al., 2009). Indeed, fast myosin heads already attached to actin substantially accelerate the further attachment of neighboring myosin heads, thereby enabling a rapid change in the rate of force development at high levels of activation (Galler et al., 2009). In contrast, slow MyHC isoforms have moderate dependence on level of activation, enabling slow CB recruitment to become faster only gradually at higher levels of activation (Galler et al., 2009). Thus, pathologic changes in myosin isoforms modify specific steps of the CB cycling as well as the overall kinetics of the CB cycle, with functional consequences on muscle performance and energy cost of contraction.

## CB alterations caused by micro-architectural changes in myofilaments

Striated muscles are characterized by a highly ordered structure that is critical for normal function and muscle homeostasis. Micro-architectural changes in myofilaments are likely contributors to muscle weakness in MDs, either directly by affecting the function of critical structural proteins, or indirectly, by increasing the activity of Ca^2+^-dependent proteases.

**Figure 3:**
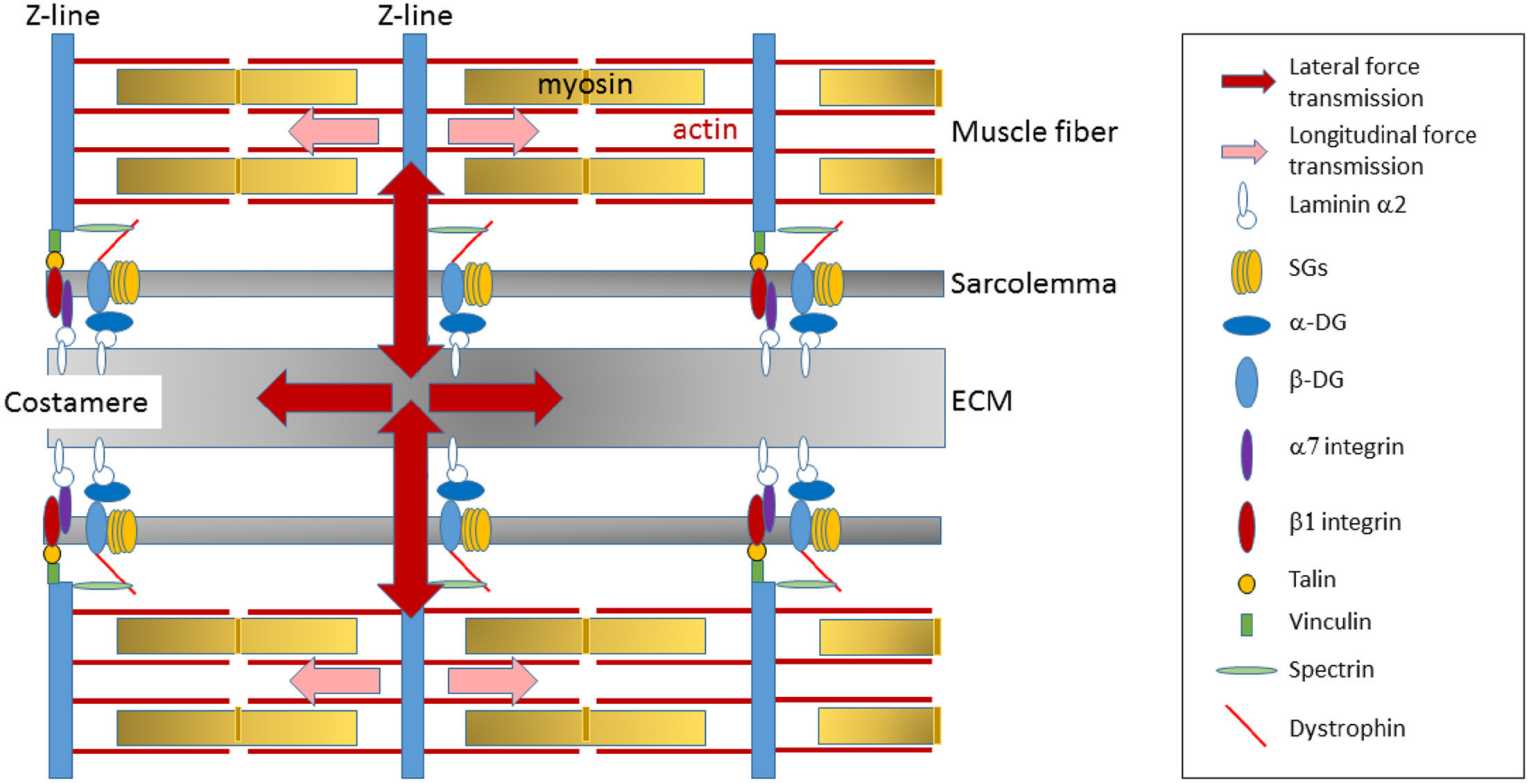
**Transmission of the forces generated in sarcomere**. The dystrophin-glycoprotein complex is preferentially located at the costamere, a protein network that reside at the sarcolemma membrane in register with the Z-lines of sarcomeres and that is critical in the lateral transmission of forces generated by the CB to the ECM and neighboring muscle fibers. Dystrophin deficiency at the lateral membrane alters costamere assembly and thus may impair the efficacy of lateral force transmission.

In striated muscle, the dystrophin glycoprotein complex is preferentially located at the costamere (Ervasti and Campbell, 1991), a protein network connecting the outermost myofibrils to the sarcolemma at each Z-disc (Pardo et al., 1983; Ervasti and Campbell, 1991; Bloch and Gonzalez-Serratos, 2003; Ervasti, 2003; Michele and Campbell, 2003). These lateral linkages are critical in the maintenance of sarcomere stability and in the transmission of forces generated by the CBs (Figure 3) (Rybakova et al., 2000; Bloch and Gonzalez-Serratos, 2003; Ervasti, 2003). Indeed, while part of the forces generated in sarcomeres is longitudinally transmitted down myofibrils in muscle to the tendon, costameres are critical to transmit the forces laterally to the ECM and neighboring muscle fibers (Street, 1983; Ervasti, 2003; Bloch et al., 2004; Ramaswamy et al., 2011). Dystrophin deficiency reduces the lateral transfer of forces between activated fibers (Ramaswamy et al., 2011), thus compromising the homogeneity of sarcomere contraction between adjacent fibers and impairing the efficacy of force transmission. As a result, each fiber tends to act as an independent longitudinal force generator, thus increasing the susceptibility of muscle membrane to contraction-induced muscle damage.

Skeletal muscle fiber contains the ubiquitously expressed and well-conserved family of Ca^2+^-dependent cysteine proteases calpains, μ-calpain, and m-calpain, as well as a muscle-specific calpain i.e., calpain-3 (Goll et al., 2003; Bartoli and Richard, 2005). Elevated calpain amount, primarily due to a significant increase in m-calpain concentration is observed in dystrophic muscles from the mdx mice (Spencer et al., 1995), a mouse model of DMD. Increased activity of calpains has been implicated in the progression of muscle wasting in dystrophic muscles (Spencer et al., 1995; Alderton and Steinhardt, 2000; Tidball and Spencer, 2000; Zhang et al., 2008). The two ubiquitous μ- and m-calpains both cleave the same substrates *in vitro*, including the troponin complex (Troponin C, Troponin I, and Troponin T), Tm, α-actinin, titin, desmin, the Z-disk protein fodrin and the sarcolemmal associated spectrin complex of proteins (Goll et al., 2003). Such substrates are consistent with a role of μ- and m-calpains in sarcomeric organization and/or dismantling. Interestingly, the two sarcomeric tropomodulin isoforms Tmod1 and Tmod4 have been recently identified as proteolytic targets of m-calpain in dystrophic muscle (Gokhin et al., 2014). Tropomodulins are dynamic actin filament pointed-end-capping proteins (Weber et al., 1994), that localize in sarcomere to each side of the M-line (Fowler et al., 1993). Absence of Tmod1 and its replacement by Tmod3 and Tmod4 in turn impair initial Tm movement over actin subunits during thin filament activation, thus reducing both the fraction of actomyosin CBs in the strongly bound state and fiber force-generating capacity (Ochala et al., 2014). Calpain-3 binds to the N2A region of titin (Sorimachi et al., 1995), but has also been identified in the nucleus of muscle cells (Baghdiguian et al., 1999). Interestingly, it has been shown that calpain-3 binding to the myofibrillar structure *in vitro* is modulated by the presence of CBs locked in rigor (Murphy et al., 2006; Murphy and Lamb, 2009), thus supporting the hypothesis that CB interactions modulate calpain-3 activity. This may explain why *in vivo*, eccentric exercise is the only physiological circumstance shown to result in the activation of calpain-3 (Murphy et al., 2007). A number of myofibrillar proteins have been identified as potential calpain-3 substrates *in vitro*, but none have been confirmed as *in vivo* targets. These include titin (at the PEVK region, adjacent to the N2A region of titin), and myosin light chain (Cohen et al., 2006). Future works are needed to determine the interrelation between calpain-3 and the CB interactions.

## Modulations and damage of CB kinetics by oxidative stress

### Definition and intracellular sources of oxidative stress in MDs

Oxidative stress has been reported as *primum movens* in both loss of cell viability and contractile dysfunction in MDs (Ragusa et al., 1997; Rando et al., 1998; Tidball and Wehling-Henricks, 2007; Menazza et al., 2010; Lawler, 2011). By definition, oxidative stress refers to a deregulation of the cellular redox-status due to an imbalance between reactive oxygen species (ROS) production and antioxidant capacities.

There are numerous potential intracellular sources of ROS in muscle tissue including the mitochondria (Murphy, 2009; Brand, 2010), the family of nicotinamide adenine dinucleotide phosphatate oxidases, now collectively known as NOX enzymes family, and a wide range of enzymes, such as xanthine oxidase, nitric oxide synthase, cyclo oxygenases, cytochrome P450 enzymes and lipoxygenases (Cheng et al., 2001). In addition, extracellular sources of ROS can arise from other non-muscle cell types, including activated neutrophils and macrophages (Moylan and Reid, 2007).

Because striated muscle cells have to adapt very rapidly and in a co-ordinated manner to changes in energy supply and oxygen flux, the control of ROS production and redox-status is tightly regulated during contraction. Importantly, the neuronal form of nitric oxide synthase (nNOS) is normally present in the sarcolemma of fast-twitch muscle fibers and associates with dystrophin (Brenman et al., 1995). Although the reaction between nitric oxide (NO) and the superoxide anion is known to form peroxynitrite, NO can also act as a protective molecule against the damaging actions of ROS (Wink et al., 1995). Accordingly, the absence of nNOS and NO in DMD muscle removes this protective action, increasing the susceptibility of DMD muscle to the damaging action of ROS (Brenman et al., 1995; Haycock et al., 1996; Disatnik et al., 1998).

### Physiological stress induces reversible modifications of proteins that contribute to normal muscle physiology

There is increasing evidence that ROS reversibly modulate important intracellular pathways involved in muscle homeostasis (Droge, 2002; Smith and Reid, 2006; Jackson, 2008; Musaro et al., 2010). Small, compartmentalized, and transient increases in ROS regulate intracellular signals by reversible oxidation of specific protein residues (Meng et al., 2002; Ghezzi, 2005; Janssen-Heininger et al., 2008; Drazic et al., 2013). Hydrogen peroxide modifies protein function by oxidizing the thiol (-SH) groups of redox sensitive cysteine residues to form disulfide bonds with adjacent cysteine residues, glutathione (glutathionylation), or small protein thiols such as thioredoxin. Reduction of the disulfide bond can then be obtained through the action of enzymes such as glutaredoxin or the peroxiredoxins (Rhee, 2006; Hashemy et al., 2007). Accordingly, specific cysteine and methionine residues can function as redox-dependent switches, thereby modulating the function of myofibrillar proteins (Nogueira et al., 2009; Mollica et al., 2012; Gross and Lehman, 2013), phosphatase or transcriptional activities (Xanthoudakis et al., 1992; Ji et al., 2004; Powers et al., 2005, 2011; Jackson, 2008; Ugarte et al., 2010). Indeed, recent findings indicate that myosin, actin, troponin I and Tm from skeletal muscle have cysteines that are critical to the effects from oxidants (Nogueira et al., 2009; Mollica et al., 2012; Gross and Lehman, 2013), and that accessibility depends on the conformation of the CB (Gross and Lehman, 2013). In addition, the function of myosin (Nogueira et al., 2009) and of the ryanodine 1 receptor (Bellinger et al., 2009) can be reversibly modified by S-nitrosylation, a redox-related modification of cysteine thiol by NO. Reversible oxidation of contractile proteins likely regulates muscle force production and actomyosin interactions, thereby contributing to the physiological adaptation of muscle to mechanical challenge (Powers and Jackson, 2008). Disturbance or removal of this redox sensitive modulations may limit the adaptability of the CB kinetics, with deleterious effects on muscle function and homeostasis (Stone and Yang, 2006; Linnane et al., 2007). Apart cysteine, methionine residue can be oxidized to its sulfoxyde, generating a mixture of two diastereoimers, denoted S and R. The S and the R forms are reduced back to methionine by methionine sulfoxyde reductase A (MsrA) and B (MsrB), respectively (Stadtman et al., 2002; Petropoulos and Friguet, 2005; Ugarte et al., 2010). Interestingly, recent studies have highlighted a new role for these Msr enzymes, identifying them as actors controlling the assembly/disassembly of actin filaments (Hung et al., 2013; Lee et al., 2013). A specific oxidation-reduction (redox) enzyme, the Mical protein, selectively modifies two methionines on the conserved pointed-end in actin filaments. Oxidized methionines at these positions simultaneously disassemble and decrease the polymerization of actin filaments (Hung et al., 2013). Recently, Hung et al. and Lee et al. showed in *Drosophila* bristle processes model and in mouse macrophages that MsrB1 stereospecifically reverses the oxidative modification of the methionines introduced by Mical, indicating that Msr system antagonizes the Mical-actin depolymerization dependent process and reversibly control actin polymerization (Hung et al., 2013; Lee et al., 2013). These data indicate new post-translational regulatory mechanisms involving oxidoreductase systems, but also open up new paths of investigation in the field of muscle diseases.

### Irreversible modifications of proteins: oxidized proteins are dysfunctional and are targeted to removal

In contrast to reversible modifications caused by low oxidative stress, chronic disruption of the oxidative balance and/or high levels of free ROS radicals cause potential biological damage, termed oxidative stress. This is a complex process that probably depends on the type of oxidant, on the site and intensity of its production, on the composition and activity of various antioxidants and on the ability of skeletal muscle to repair damaged fibers. Irreversible damage of myofibrillar proteins may affect the CB cycle, as observed after nitrosylation and carbonylation of myosin (Coirault et al., 2007; Guellich et al., 2007), and target them for catabolic proteolysis (Jung et al., 2007). Alternatively, oxidative stress can activate caspases that in turn cleave myofibrillar proteins and impair CB properties (Du et al., 2004).

## Functional consequences of altered CB cycling on muscle contraction

The functional consequences of altered CB cycle in MD remain to be precisely determined. It has been reported that the reduced muscle strength per cross section in *mdx* diaphragm is associated with an accelerated cycle compared with control diaphragm muscle (Coirault et al., 1999). Assuming that one molecule of ATP is hydrolyzed per CB cycle (Huxley, 1957; Huxley and Simmons, 1971; Eisenberg et al., 1980), these data suggested that the overall cycle of ATP splitting takes place more rapidly in *mdx* than in control mouse diaphragm, thus reducing the efficiency of muscle contraction to sustain force. Overall, these data suggested that absence of the sarcolemmal dystrophin protein leads to damaged or dysfunctional myosin molecules that impair CB kinetics and may account for the contractile deficit in *mdx* diaphragm. However, while mechanical differences are observed between *mdx* and control diaphragms at the intact muscle strip level, these differences are not observed at the single permeabilized cell (Bates et al., 2013). In addition, it was reported that myosin extracted from bulk *mdx* mouse diaphragm muscle moves actin filaments in an “*in vitro* motility assay” at a lower velocity than myosin extracted from controls (Coirault et al., 2002). Consistent reduction in actin sliding velocity has been found in pure MyHC-2 isoform from mdx gastrocnemius muscle, but not in type 1 myosin from wild-type and *mdx* muscle (Canepari et al., 2009). Therefore, observed changes in myosin velocity have been related to a change in the intrinsic properties of the molecule, but was not attributed to a change in the proportion of different myosin isoforms in the sample. In addition, it has been shown that nebulin in skeletal muscle increases thin filament activation via increasing CB cycling kinetics leading to an increased force and efficiency of contraction (Chandra et al., 2009). Results from that study provide novel insights regarding nebulin-based nemaline myopathy. Thus, complex mechanisms other than translational changes in myosin isoform may contribute to CB modifications in muscular disorders. Future studies are needed to precisely determine the contribution of post-translational modifications of contractile proteins to muscle weakness in MDs.

## Conclusions and future directions

The mechanisms of muscle weakness have been a fundamental question of the physiopathology of muscle dystrophy for more than 50 years. It is now clear that the molecular causes responsible for the impaired performance in MDs involve both structural and functional modifications of the acto-myosin interactions. The precise mechanisms are complex with probably some specificities related to each individual mutation responsible for the MD, the severity of the translational and post-translational alterations in myofibrillar proteins, and on the ability of skeletal muscle to adapt to these changes. Whereas the functional consequences of translational changes in contractile proteins have been extensively studied, the impact of post-translational changes on the function of various contractile proteins still remains to be precisely assessed in the context of MDs. This is a particularly exciting time to study this question as recent findings strongly suggest that the loss of transient structural modifications of the contractile proteins such as reversible oxidation of specific protein residues impair the ability of CBs to adapt very rapidly to changes in workload and energy supply. Future works will assess the impact of non-permanent structural modifications of actin, myosin, and regulatory proteins on muscle performance and determine their potential contribution to muscle weakness in MDs.

### Conflict of interest statement

The authors declare that the research was conducted in the absence of any commercial or financial relationships that could be construed as a potential conflict of interest.

## Abbreviations

ADP: adenosine 5′-diphosphate
ATP: adenosine 5′-triphosphate
CB: cross-bridge
DMD: Duchenne muscular dystrophy
ECM: extracellular matrix
FSHD: fascioscapulohumeral muscular dystrophy
MD: muscular dystrophy
MsrA and MrB: methionine sulfoxyde reductase A and B
MyHC: myosin heavy chain
nNOS: neuronal NO synthase
NO: nitric oxide
Pi: inorganic phosphate
ROS: reactive oxygen species
Tm: tropomyosin.
